# Modeling of *Valeriana wallichii* Habitat Suitability and Niche Dynamics in the Himalayan Region under Anticipated Climate Change

**DOI:** 10.3390/biology11040498

**Published:** 2022-03-24

**Authors:** Priyanka Kumari, Ishfaq Ahmad Wani, Sajid Khan, Susheel Verma, Shazia Mushtaq, Aneela Gulnaz, Bilal Ahamad Paray

**Affiliations:** 1 Conservation and Molecular Biology Laboratory, Department of Botany, Baba Ghulam Shah Badshah University Rajouri, Jammu and Kashmir 185234, India; pk838511@gmail.com (P.K.); waniishfaq680@gmail.com (I.A.W.); sajidkhan717@gmail.com (S.K.); 2 Department of Botany, University of Jammu, Jammu and Kashmir 180001, India; 3 Department of Botany, Sri Pratap College, Jammu and Kashmir 190001, India; shaazia23@gmail.com; 4 College of Pharmacy, Woosuk University, Wanju-gun 55338, Korea; draneela@woosuk.ac.kr; 5 Department of Zoology, College of Science, King Saud University, Riyadh 11451, Saudi Arabia; bparay@ksu.edu.sa

**Keywords:** BIOMOD ensemble approach, distribution modeling, range change, rewilding, species recovery, natural habitats

## Abstract

**Simple Summary:**

In the modern era of the anthropocene, an increase in the accumulation of greenhouse gases in the atmosphere has resulted in the global rise of the Earth’s temperature. This has resulted in the habitat shift of various plant and animal species. Plant species that are endemic show a narrow distribution range and inhabit higher elevations are highly susceptible to the impacts of global warming. The present study discusses the habitat distribution of *Valeriana wallichi*, an endangered medicinal herb. We also predicted the impact of climate change on its distribution range and niche dynamics. Results reveal significant contraction in the possible habitats of this herb under future climatic scenarios, with RCP 8.5 for 2070 showing the highest habitat loss. Niche equivalency and similarity tests describe that the habitats could be compared but not identical between current and future climatic scenarios. From the current study, we conclude that the habitats of *Valeriana wallichii* are highly vulnerable to climate shifts. This study can not only be used to alleviate the threat to this plant by documenting the unexplored populations, restoring the degraded habitats through rewilding and launching species recovery plans in the natural habitats but could also be used by various conservation biologists to recover the declining status of various highly valued species across the globe.

**Abstract:**

An increase in atmospheric greenhouse gases necessitates the use of species distribution models (SDMs) in modeling suitable habitats and projecting the impact of climate change on the future range shifts of the species. The present study is based on the BIOMOD ensemble approach to map the currently suitable habitats and predict the impact of climate change on the niche shift of *Valeriana wallichii*. We also studied its niche dynamics using the ecospat package in R software. Values of the area under curve (AUC) and true skill statistics (TSS) were highly significant (>0.9), which shows that the model has run better. From 19 different bioclimatic variables, only 8 were retained after correlation, among which bio_17 (precipitation of driest quarter), bio_1 (annual mean temperature), and bio_12 (annual mean precipitation) received the highest gain. Under future climate change, the suitable habitats will be significantly contracted by −94% (under representative concentration pathway RCP 8.5 for 2070) and −80.22% (under RCP 8.5 for 2050). There is a slight increase in habitat suitability by +16.69% (RCP 4.5 for 2050) and +8.9% (RCP 8.5 for 2050) under future climate change scenarios. The equivalency and similarity tests of niche dynamics show that the habitat suitability for current and future climatic scenarios is comparable but not identical. Principal Component Analysis (PCA) analysis shows that climatic conditions will be severely affected between current and future scenarios. From this study, we conclude that the habitats of *Valeriana wallichii* are highly vulnerable to climate shifts. This study can be used to alleviate the threat to this plant by documenting the unexplored populations, restoring the degraded habitats through rewilding, and launching species recovery plans in the natural habitats.

## 1. Introduction

The ever-growing increase in the concentration of greenhouse gases has threatened the existence of different plant species and has altered the function and stability of different ecosystems across the globe. However, the fragile landscapes within the Himalayan biodiversity regions show greater susceptibility to the climate change scenarios and therefore elevate the ongoing concern about impacts on the biodiversity in these regions [1,2,3,4,5]. The major consequence of climate change is habitat range shifts at species or ecosystem level, which affects natural selection, generates co-evolutionary interactions, and destabilizes the ecosystem functional traits. Various model projections generated by predicting the impact of climate change reveal that gradient-based mountain ecosystems receive the consistent upward shift of the species and could be the most vulnerable of all the terrestrial ecosystems [6,7,8]. It is predicted that on account of global warming due to increased Earth temperature and change in precipitation pattern, suitable habitats of several high mountain plant species would be drastically reduced or will disappear by the end of the twenty-first century [9,10,11].

In the contemporary world of the anthropocene, the impact of human-mediated ecological and socio-economic changes (anthropogenic activities, infrastructure development, climate change, and alien species invasion) [12,13] are deep and complex; however, the effects of these on ecosystems in the majority of cases, remain unpredictable [14]. Mapping ecological niches and anticipating the influence of global warming on the potential habitat of endangered plant species is extremely important for monitoring, management, and rehabilitation of their dwindling populations and natural habitats [15]. Habitat distribution modeling help to forecast the distribution range, especially the realized niche, by using the information records obtained through extensive field surveys in concurrence with topographic and bioclimatic variables [16,17]. Threatened and endemic species, which are ecological specialists and show smaller geographic distribution range, small population size, and low reproductive capacity, are highly vulnerable to such alterations and are at greater risk of extinction [18,19]. Thus, their conservation should be prioritized and accomplished through the application of different ecological principles within or near their natural habitats [20].

The use of several species distribution models (SDMs) in forecasting habitat suitability has become an important ecological tool [19,21]. SDMs have been widely used in restoring degraded habitats [22,23,24,25], introduction of native germplasm, or selecting appropriate sites for species conservation and management [26,27,28]. Recent developments in SDMs show diverse implications to solve the conservation-related challenges by generating suitable niche models and predicting range estimates [29], network design and prioritization of protected areas [30,31], and projecting the impact of climate change on reproduction and future distributions [25,31]. SDMs combine species occurrence data with ecological/environmental variables (temperature, precipitation, elevation, geology, and vegetation) to create a model that represents the distribution of species with respect to different environmental variables [26]. The application of the BIOMOD ensemble approach holds a rational stride in species distribution modeling, as it makes the combined use of nine different algorithms. It evaluates the species distribution pattern and its potential alterations under future climate changes [32,33,34]. To optimize the prediction of habitat suitability, BIOMOD incorporates a variety of statistical and machine learning methodologies [35,36].

*Valeriana wallichii* is an evergreen herb. It emits a pleasant odor, due to which this species derives its local name “Mushkbala”. The sporophytic body consists of an underground rhizome and leafy offshoots. With an onset of the flowering peduncles arise from the offshoots. The flowering season extends from the second to third week of May. Peak flowering time is from February to March [37]. It is a highly valued medicinal and aromatic shrub having immense use in the folk medicine and pharmaceutical sector and is extensively used as a sedative, stomachic, anti-spasmodic, nervine, stimulant, carminative, and analeptic [37]. Incessant over-exploitation of this species has led to the progressive dwindling of its natural populations, and as such, it has been listed among threatened species by IUCN [37]. Modeling the current suitable areas and anticipating the influence of climate shifts on habitat suitability in future climatic scenarios can be an important ecological tool to restore the diminishing populations of this Himalayan angiosperm. In the current study, we used the BIOMOD ensemble approach to:

(i) Study the role of different bioclimatic variables on the habitat distribution of *V. wallichii*;
(ii) Model the current distribution range of *V. wallichii* in the Himalayan biodiversity hotspots;
(iii) Model the climate change-driven shifting patterns in the distribution of *V. wallichii* at different spatiotemporal scales;
(iv) Predicting the extent and rate of potential range expansion/contraction of *V. wallichii* and evaluating the niche dynamics using the models generated to formulate different management strategies.

## 2. Materials and Methods

### 2.1. Distribution Data

Extensive field surveys were conducted from the northwestern and western mountain ranges of the Indian Himalayas. A total of 42 new populations were inventoried from the Jammu and Kashmir, Ladakh, and Uttarakhand regions of India (Table 1). Their geographical coordinates were recorded with the help of GPS (Magellan eXplorist 30H and Magellan Professional Mobile Mapper (990603-50)). The distribution data were further supplemented with data obtained from the GBIF portal (Global Biodiversity Information Portal) and Botanical Information and Ecology Network (BIEN) (accessed on 18 April 2021). A total of 276 geo-referenced presence records were obtained and were reduced to 51 occurrence points after clipping for the study area (i.e., Himalaya). Each of the occurrence records was thoroughly checked for accuracy before usage. A total of 37 geo-referenced points were retained after spatial thinning and used for modeling the habitat distribution of *Valeriana wallichii*.

### 2.2. Bioclimatic Variables and Their Importance

For modeling the habitat distribution of *V. wallichii*, nineteen different bioclimatic variables with 30 arc seconds spatial resolution data were obtained from Worldclime data set v1.4 (https://www.worldclim.org accessed on 21 May 2021). As bioclimatic variables are highly subjected to auto-correlation, we performed Pearson’s correlation among various bioclimatic variables. Those variables that depict the coefficient of correlation (r) values higher than 0.75 were simplified and reduced to one variable. After correlation analysis of 19 bioclimatic variables, only eight were retained and used for modeling the distribution of *Valeriana wallichii* (Table 2 and Table 3).

The relative influence of each bioclimatic variable in determining the distribution of selected plant species, we employed the permutation approach [36]. Predictions are created using a specific algorithm after changing only one target variable while the rest of the variables are maintained fixed in this approach. The variable importance estimates are calculated as one minus correlation score (1—correlation score) between the original prediction and the prediction made with a permuted variable [2]. As a result, high values show that the predictor variable is more relevant in the model, while a value of 0 denotes that the variable has no significance in the model.

### 2.3. Modeling Technique

Nine different algorithms implemented in the biomod2 package integrated within R statistical software (v 4.0.3; R Development Core Team 2021) were used to perform species distribution modeling and ensemble forecasting. These modeling algorithms work on the basis of both absence and presence data sets; however, it is difficult to obtain the actual absence data. We generated 10,000 pseudo-absences within the study area following Barbet-Massin et al. [38] and Guisan et al. [39]. The models were created with training sets of 80% and 20% data for the validation set. We repeated the method three times in total, yielding 27 models for each time period and climate scenario. The model’s performance was assessed using two types of assessment metrics: the area under the curve (AUC) of receiver operating characteristics (ROC) and true skills statistics (TSS) [40,41]. AUC values range from 0 to 1, with 0.5–0.7 suggesting poor model performance, 0.7–0.9 indicating acceptable model performance, and >0.9 indicating outstanding model performance [42].

Similarly, the TSS measure ranges from −1 to +1, with TSS values below 0.40 suggesting poor model performance, 0.40 to 0.75 indicating suitable model performance, and 0.75 and above indicating higher model performance [40,43]. We constructed the final ensemble model for each climatic scenario and time period combination using committee averaging and weighted mean approaches individually [41]. To generate the final ensemble models, we only maintained models with TSS scores larger than or equal to 0.8. As a result, we obtained a total of five ensemble predictions for current climatic suitability, and four future predicted habitat suitabilities for the two time periods (2050 and 2070), reflecting two concentration paths (RCP 4.5 and 8.5).

### 2.4. Species Range Change

We used the BIOMOD-Range Size function in the biomod2 package to quantify and represent the range change over future climatic scenarios for *Valeriana wallichii*. This production function exhibits two outputs: A table containing a brief description related to statistics of species range change and a spatial map that shows the probable areas where the species will show habitat gain or loss in the future climate scenarios. “Gain” corresponds to the number of pixels that are predicted to be occupied by species under future climate scenarios and currently not occupied by the studied species; “Loss” shows the number of pixels anticipated to be lost by the studied species, “Stable” denotes the number of pixels currently occupied by the studied species while “Absent” represents the number of pixels that are not currently occupied by the studied species, “Percentage loss” was calculated as (Loss/(Loss + Stable)); “Percentage gain” was determined by using the formula (Gain/(Loss + Stable)) and “Range change” was calculated by percentage gain—percentage loss.

### 2.5. Niche Overlap

Modified principal component analysis (PCA-env) was used for determining the niche overlap of the plant species under current and future climatic scenarios [44]. Bioclimatic variables are changed into two-dimensional space defined by two principal components. The two-dimensional environmental space is then projected onto grid cells with a diameter of 100 × 100 and bounded by minimum and maximum PCA values in the background. Smooth key density function was used to overcome sampling bias due to the lower number of occurrence data points [45]. Schoener’s D metric was used to determine the extent of niche overlap. It varies from 0, representing no overlap, to 1, representing complete overlap. In order to understand the importance of niche overlap in the geographic area, niche equivalency and similarity tests were performed [44]. Niche equivalence test was performed by the comparison of niche overlap (D) values for current and future climatic scenarios and comparing it to the overlap of the null distribution. If the overlap values are significantly lower than niche values, then the null hypothesis of niche equivalency is rejected [44]. The niche similarity test determines if the niches of two entities being examined are more or less similar than projected by chance. It also takes into account the bioclimatic variables of the background space across the study area. [46]. The niche analysis was carried out by using the R software-based package “ecospat” [47].

## 3. Results

### 3.1. Model Evaluation and Variable Contribution

**Figure 1:**
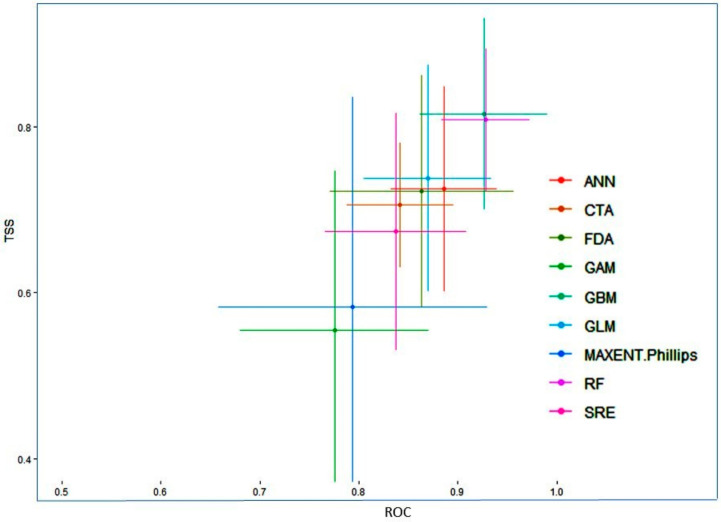
True skill statistics (TSS) and receivers operating characteristic curve (ROC) values of different model algorithms.

The present study suggests that the final ensemble model produced for *Valeriana wallichii* in terms of committee averaging had AUC values equal to 0.99 and TSS values equal to 0.93. Likewise, in terms of weighted means, the ensemble models produced had AUC values equal to 0.99 and TSS values equal to 0.92. The values in terms of committee averaging and weighted means demonstrate that the final model is robust in predicting the distribution of the *Valeriana wallichii*. While comparing the predictive accuracy at the individual algorithm level, the model showed GBM, RF, GLM showed the highest accuracy, followed by ANN, FDA, MAXENT Phillips, CTA and SRE (Figure 1).

The selected bioclimatic variables showed variations across different algorithms in *Valeriana wallichii*. The bioclimatic variables that were highly influential and represented the highest gain in determining the potential habitat distribution of the species were precipitation of driest quarter (bio_17), having importance scores that range from 0.06 to 0.82 followed by annual mean temperature (bio_01) with importance scores ranging from 0.06 to 0.80 (Table 4).

### 3.2. Current and Future Habitat Distribution

**Figure 2:**
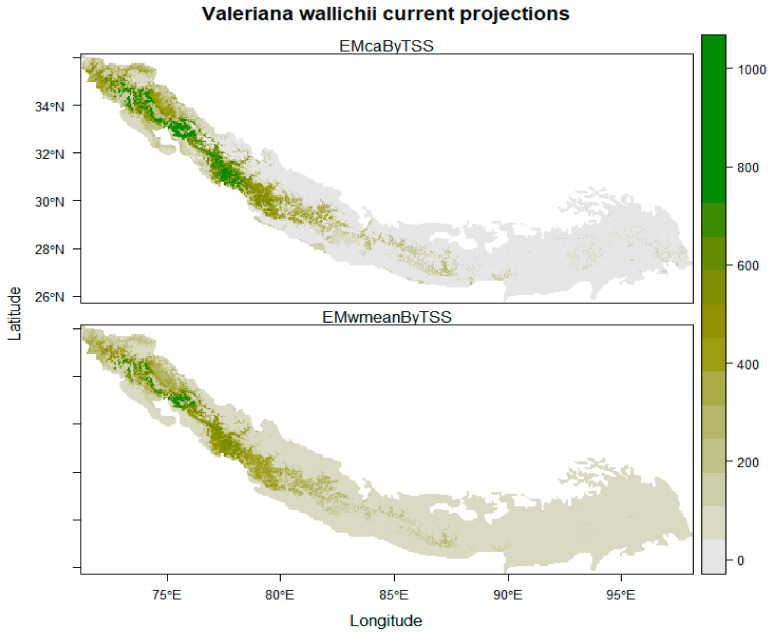
Current distribution pattern of *Valeriana wallichii* in Himalayan biodiversity hotspots. (The scale from 0 to 1000 represents the habitat suitability class where 0 depicts the absence of the species, 200–400 show least suitable areas, 400–600 show marginally suitable areas, 600–800 show moderately suitable areas, while 800–1000 show highly suitable areas).

**Figure 3:**
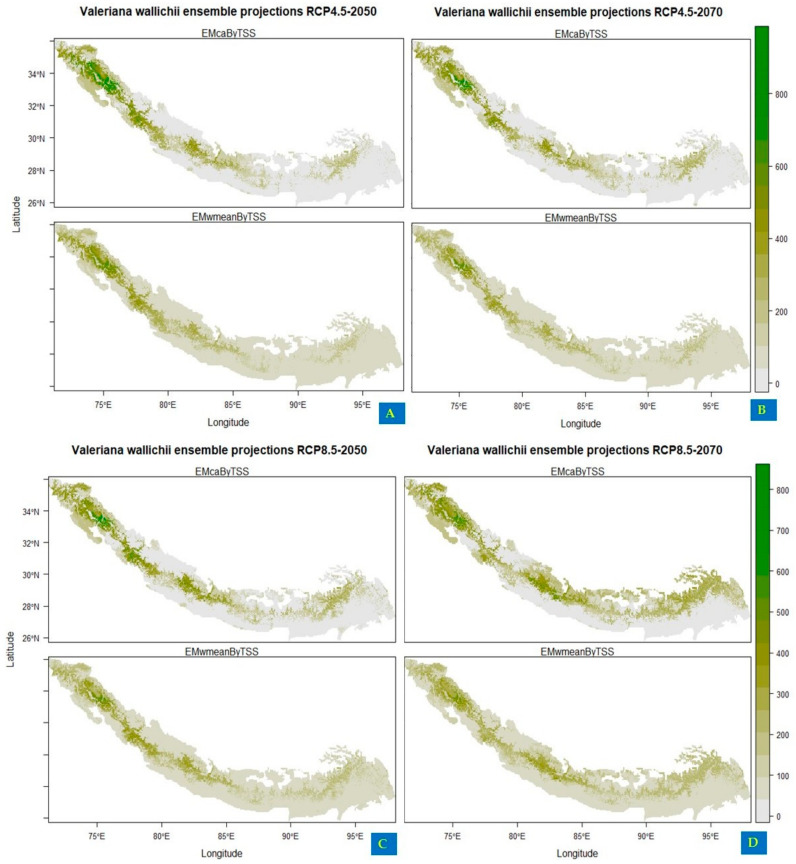
Future habitat distribution of *Valeriana wallichii* under projected climate change (**A**,**B**): showing the impact of climate change under representation concentration pathway 4.5 for two time periods, i.e., 2050 and 2070 (**C**,**D**): showing the impact of climate change under representation concentration pathway 8.5 for two time periods, i.e., 2050 and 2070.

Under current climatic conditions, the ensemble model showed that the northwestern regions of India (Jammu and Kashmir, Uttarakhand and Himachal Pradesh), northwestern areas of Pakistan (Khyber Pakhtunkhwa, Swat valley and Muzaffarabad), and southwestern parts of Nepal (Dhangadhi, Silgarhi, Tapoban) show highly suitable areas for the growth of *Valeriana wallichii* whereas southwestern parts of Nepal shows moderately low habitat suitability. In addition, the southeastern parts of Bhutan and Sikkim show moderately low habitat suitability, and southeastern parts of Arunachal Pradesh and northwestern parts of Assam show low suitability areas for the growth of *Valeriana wallichii* (Figure 2). Future climate change scenarios for *Valeriana wallichii* predicted that there would be a significant decrease in habitat suitability, and this plant species will show a significant contraction in their possible suitable habitats. Although some of the currently suitable areas continually will remain suitable under future climatic conditions also. These regions include most of the western Himalayas, i.e., the northern part of Pakistan, northwestern part of Jammu and Kashmir, Himachal Pradesh and Uttarakhand, the southern part of Nepal, Bhutan, and Sikkim. Some areas such as the southeastern parts of Arunachal Pradesh and northwestern parts of Assam show moderate to low habitat suitability under RCP 4.5, and high suitability under RCP 8.5 but are currently unsuitable and are predicted to be suitable under future climatic conditions (Figure 3A–D).

### 3.3. Species Range Shift under Future Climatic Conditions

**Figure 4:**
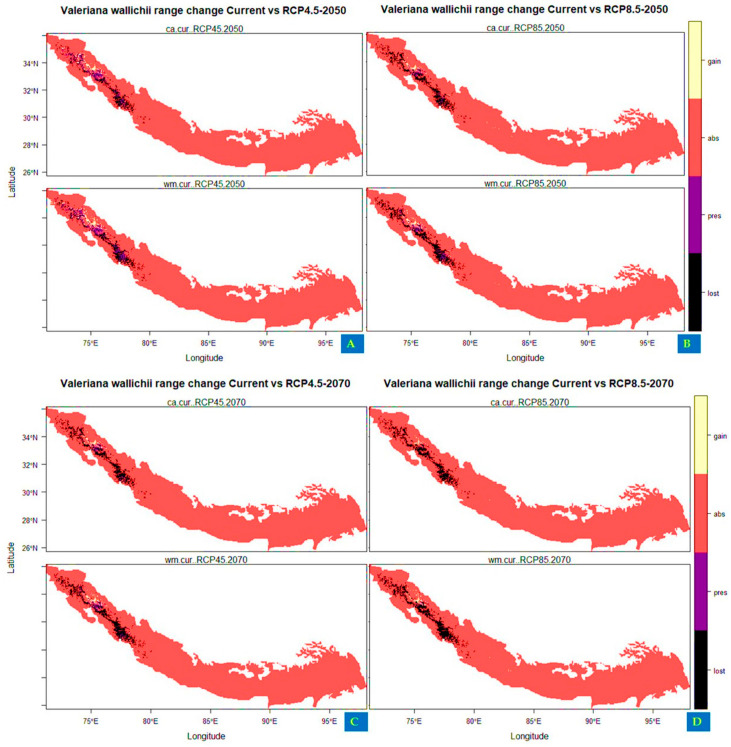
Distribution maps showing range change area of *Valeriana wallichii* under future climatic scenarios (**A**,**B**): under representative concentration pathway 4.5 and 8.5 for year 2050 (**C**,**D**): under RCP 4.5 and 8.5 for year 2070. Olive color in the scale represents the areas where newly suitable areas appear in the future climate scenarios, black color represents the areas where the habitat suitability is lost in the future, purple color represents the regions that maintain the habitat suitability in the future climatic conditions, and red color shows the regions where the species is absent.

The projections for future habitat suitability of *Valeriana wallichii* displayed that the species will undergo severe range contraction under future climatic scenarios. Specifically, by assuming its dispersal throughout Himalayan biodiversity hotspots, the proportion to its current suitable habitats is projected to be lost under future climate scenarios. The suitable habitat of *Valeriana wallichii* could be reduced by about 57.231% (under RCP4.5, 2050), 88.251% (under RCP4.5, 2070), 89.178% (RCP8.5, 2050), and by about 97.878% under RCP8.5 for the year 2070 when compared to current habitat suitability (in terms of committee averaging). In terms of weighted mean, the suitable habitat will be reduced by about 54.123% (under RCP4.5, 2050), 84.765% (under RCP4.5, 2070), 81.998% (under RCP8.5, 2050), and by about 97.050% under RCP8.5 for the year 2070. The areas that are likely to become unsuitable in the future are located mostly toward northwestern parts of Nepal, eastern parts of Uttarakhand, some areas of Himachal Pradesh, and Jammu and Kashmir. In comparison to this, the currently unsuitable areas become suitable under future climate scenarios with a range expansion in terms of committee averaging of 16.692% (under RCP4.5 2050), 8.564% (under RCP4.5 2070), 8.953% (under RCP8.5 2050), and 3.808% (under RCP8.5 2070) (Table 5). Likewise, some of the currently unsuitable areas become suitable under future climate in terms of weighted mean with a range expansion of 18.545% (under RCP4.5 2050), 9.630% (RCP4.5 2070), 10.080% (RCP8.5 2050), and 6.352% (under RCP8.5 2070) and include mainly the northern part of Pakistan, northern parts of Jammu and Kashmir, Himachal Pradesh, Uttarakhand and western parts of Nepal. (Figure 4A–D).

### 3.4. Niche Dynamics

**Figure 5:**
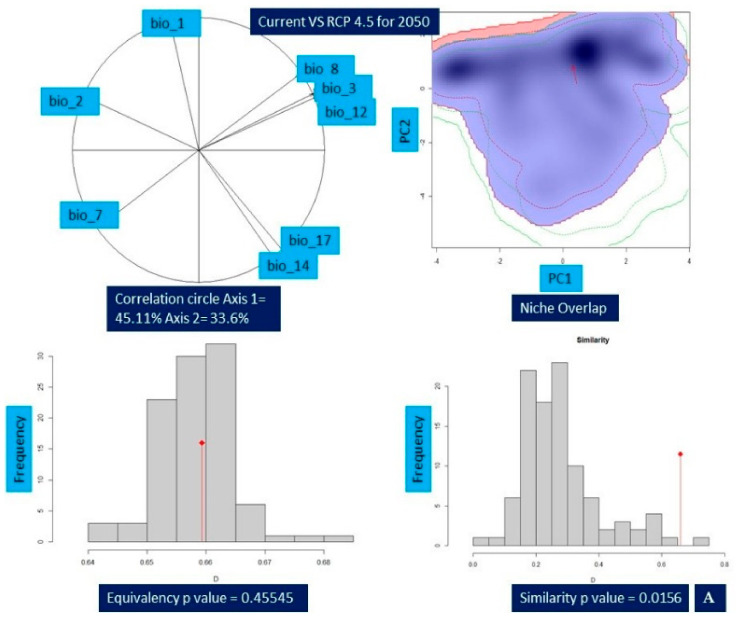

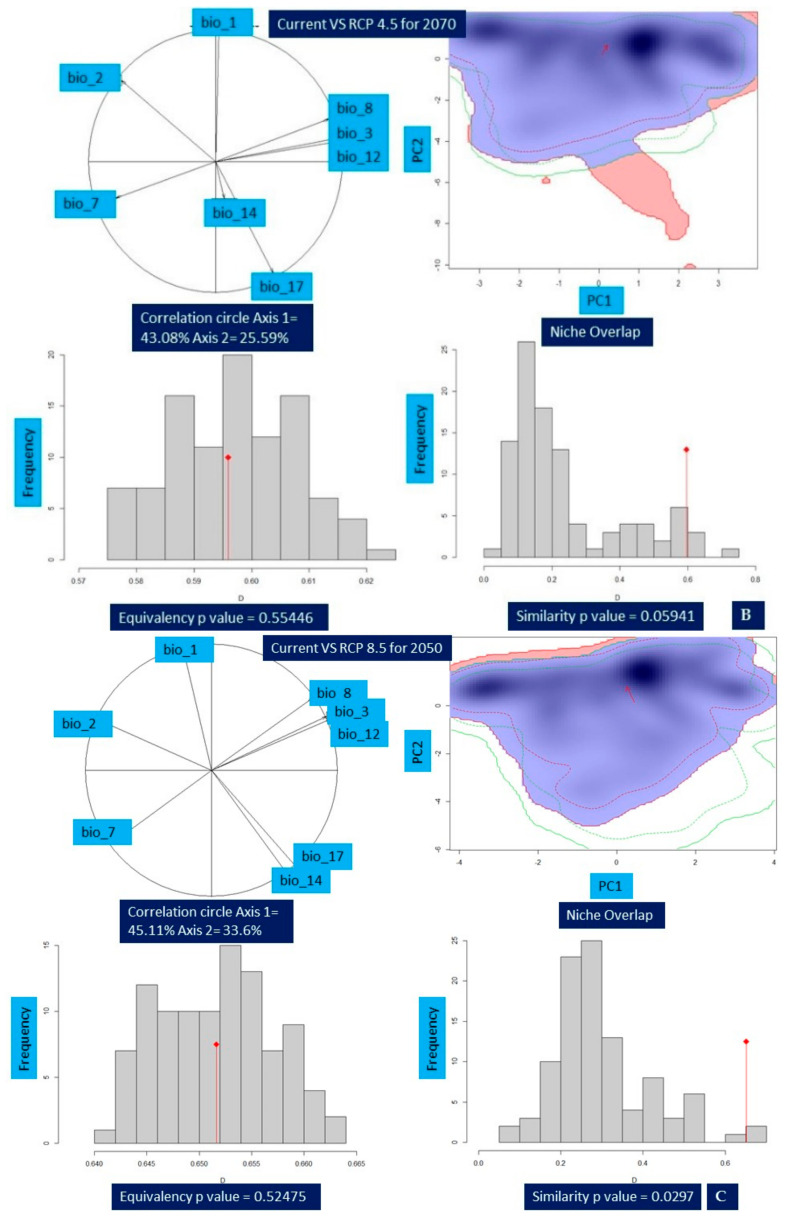

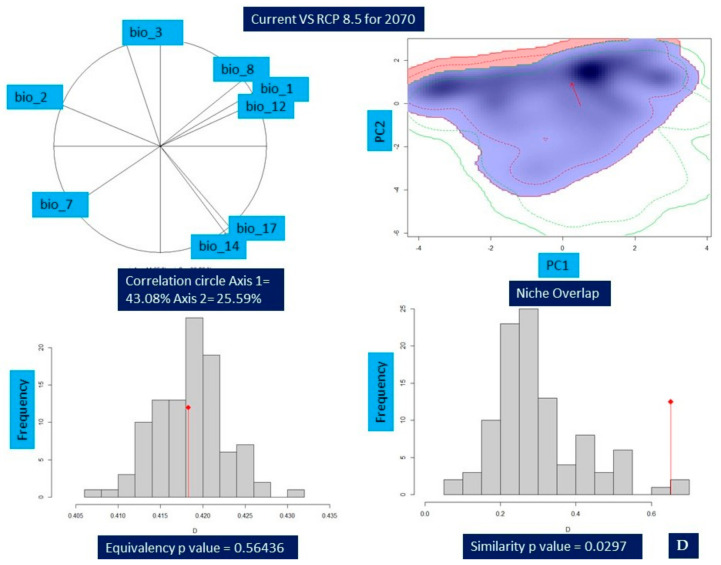
Change in the niche dynamics of *Valeriana wallichii* under current and future climatic scenarios. (**A**) Current vs. representative concentration pathway 4.5 for 2050 (**B**) Current vs. representative concentration pathway 4.5 for 2070 (**C**) Current vs. representative concentration pathway 8.5 for 2050 (**D**) Current vs. representative concentration pathway 8.5 for 2070. The contribution of various bioclimatic variables is provided by correlation circle and depicts the variation percentage of each axis. Blue color represents the density of species in current while red color shows the density of target species in future. Equivalency and similarity results are shown by bar plots, while the red arrow shows the change in the niche between current and future climate scenarios.

Niche comparison results of *Valeriana wallichii* between the current and future climatic unveiled that niche overlap ranged from 42% (Schoener’s D = 0.42 (min)) for current vs. RCP8.5 2070 to 66% (Schoener’s D = 0.66 (max)) for current vs. RCP4.5 2050. In addition, an overlap of 60% for current vs. RCP4.5 2070 (Schoener’s D = 0.60) and overlap of 65% for current vs. RCP8.5 2050 (Schoener’s D = 0.65) were found, respectively. While measuring the properties of environmental habitat, the principal component analysis (PCA-env) revealed that principal component 1 (PC1) retained a maximum variation of 45.76% in the case of current vs. RCP 8.5-2050 in comparison to the current vs. RCP4.5 2070, which retained a minimum variation of 43.08%. Similarly, the minimum variation retained for principal component 2 (PC2) is 25.59% for current vs. RCP4.5 2070 compared to a maximum of 33.6% for current vs. RCP4.5 2050. For niche equivalency, the null hypotheses for each of the pairwise comparisons between the species environmental niche were not rejected for current and future climatic scenarios in any of the pairwise comparison, i.e., *p* > 0.05. Niche similarity test was rejected for the null hypothesis in case of current vs. RCP4.5 2050 (*p*-value = 0.198), current vs. RCP8.5 2050 (*p*-value = 0.0297), current vs. RCP8.5 2070 (*p*-value = 0.0495) respectively (Table 6; Figure 5A–D).

## 4. Discussion

Modeling current habitats and forecasting the future species distribution range is an important step toward the species recovery plans in their natural habitats. The use of habitat distribution modeling to determine the dispersal range and impact of climate change on future habitat suitability is crucial to devise appropriate management practices for the conservation and sustainability of the habitat of species in the future [48,49]. This not only provides the key determinants of geographical range area [50] but also enables us to understand the interrelationship among species distribution range and extinction [51,52]. Over the past several decades, increased global Earth temperature has resulted in the extinction of a wide range of plant species [53,54], while a large number show shifts in their ecological niches [53,55]. The impacts of climate change are experienced by all types of ecosystems across the globe; however, the uphill habitats within the Himalayan biodiversity hotspots are highly vulnerable to the potential climate change impacts, the prime concern among which is the alteration in habitat suitability and niche expansion/contraction [56,57]. The present study is the first effort to map the current habitats and predict the impact of climate change on habitat suitability in the future using the BIOMOD ensemble approach in *Valeriana wallichi*, a threatened medicinal herb. BIOMOD ensemble was preferred over a single algorithm modeling data set because it combines different machine learning and statistical algorithms to increase the accuracy of the model run estimations [58,59]. To determine the performance of the model run, two types of evaluation metrics were used, AUC and TSS [60,61]. In the present study, the values of both these metrics were greater than 0.9. Moreover, the actual geographical distribution recorded during the present study coincides with the current potential habitats modeled in the present study, which signifies the robustness of the model run.

While modeling the endemic and threatened plant species, the application of an appropriate number and combination of bioclimatic variables is necessary [62,63,64,65]. Bioclimatic variables are not the sole determinants of the ecological niches. Different topographic and edaphic factors also interact with the biotic components of the ecosystem and show a significant impact on their distribution range [66,67]. However, when the modeling of species is performed in larger geographical areas, climatic conditions significantly determine habitat suitability [68]. Our study predicted that both precipitation (bio_17) and temperature (bio_1)-based variables show a greater impact on the habitat suitability of *Valeriana wallichii*. These results are supported by the findings of other workers who have reported a significant contribution of different precipitation and temperature-based climatic variables in governing the habitat distribution of different Himalayan plants [69,70,71,72]. Both precipitation and temperature-based bioclimatic variables are vital for the development and morphogenesis of the *Valeriana wallichii* because it is an evergreen herb. Precipitation maintains the available moisture within the soil for rhizome, while the temperature-based variable is important in the growth and development of aerials shoots.

In India (Jammu and Kashmir, Himachal Pradesh, Uttarakhand), northwestern areas of Pakistan (Khyber Pakhtunkhwa, Swat valley and Muzaffarabad) and southwestern parts of Nepal (Dhangadhi, Silgarhi, Tapoban) show highly suitability areas for the growth of *Valeriana wallichii,* whereas southwestern parts of Nepal shows moderately low habitat suitability. The greater distribution range of *Valeriana wallichii* toward the western and northwestern regions could be due to the availability of sub-alpine regions with greater habitat suitability. In addition, southeastern parts of Bhutan and Sikkim show moderately low habitat suitability, and southeastern parts of Arunachal Pradesh and northwestern parts of Assam show low suitability areas for the growth of *Valeriana wallichii*. These predictions are supported by the results of different workers who reported a similar kind of distribution pattern for high-altitude medicinal plants of Himalayan regions [73,74]. The habitat suitability of the species in fragile Himalayan landscapes is extremely vulnerable to the impacts of climate change [56]. For the future climate change scenarios under RCPs 4.5 and 8.5 (2050 and 2070), our ensemble model indicated that there occurs a significant contraction in the habitat suitability of these plant species. The large area from current suitable habitats will become less or not suitable in the future, and some regions with climatically less or not suitable areas will show higher climatic suitability. These observations are in agreement with the results of Wei et al. [6], who also reported similar kinds of range change for other medicinal herbs in the alpines of Himalaya. Future climatic scenarios show a significant contraction of suitable habitats under all RCPs and time periods. Northwestern Himalayas receive higher climate change trends as compared to eastern Himalayas [75,76,77]. Under the increased global Earth temperature, the precipitation of the driest quarter will be significantly reduced, which will ultimately lead to the fluctuations in the annual mean temperature, the two main determinants in the possible distribution of *Valeriana wallichii*. Significant habitat contraction in the northwestern and western Himalayas could be attributed to the change in these climatic variables. Under the future climatic projections, the likelihood of these plant species to move and inhabit the areas that lie at greater elevations may reflect the niche shift of the species due to the projected increase in the Earth’s temperature in the future. These predictions can be related to the modeling studies carried out by various researchers on different Himalayan plant species [78,79]. Based on these findings, it is clear that species may face comparable but not identical environmental circumstances throughout current and future climate forecasts [80,81,82].

## 5. Conclusions

The current study is the first attempt to study the impact of climate change on habitat distribution and niche dynamics of *Valeriana wallichii*, a highly valued medicinal herb. The outcome of the present study credence to the SDMs’ anticipated outcomes, indicating that the current highly appropriate habitat will be constricted in the future while expanding to other areas of the study area that are currently less suited. The evaluation of the niche equivalence test clearly shows that the species’ environmental niche will not remain precisely the same under current and future climatic scenarios. Similarly, the niche similarity test indicated a considerable degree of overlap between the species under present and future environmental circumstances.

## Figures and Tables

**Table 1:** Geographical coordinates and altitude of different study areas.

Site No.	Site	Coordinates	Altitude
1	Manyal Gali, J&K	33°33′ N 74°22′ E	1903 m.a.s.l
2	Dera Ki Gali, J&K	33°35′ N 74°21′ E	2126 m.a.s.l
3	Bafliaz, J&K	33°21′ N 74°21′ E	1566 m.a.s.l
4	Noorichamb, J&K	33°36′ N 74°25′ E	1834 m.a.s.l
5	Bakori, J&K	33°21′ N 74°31′ E	1637 m.a.s.l
6	Budhal, J&K	33°22′ N 74°38′ E	1781 m.a.s.l
7	Patnitop, J&K	33°05′ N 75°19′ E	2072 m.a.s.l
8	Batote, J&K	33°01′ N 75°39′ E	1656 m.a.s.l
9	Amiranagar, J&K	33°00′ N 75°05′ E	1498 m.a.s.l
10	Neota, J&K	33°02′ N 75°03′ E	1327 m.a.s.l
11	Drudhoo, J&K	33°15′ N 75°45′ E	1366 m.a.s.l
12	Nai Basti, J&K	33°01′ N 75°39′ E	1370 m.a.s.l
13	Dranga, J&K	33°01′ N 75°40′ E	1383 m.a.s.l
14	Narnoo 1, J&K	33°0′ N 75°40′ E	1378 m.a.s.l
15	Narnoo 2, J&K	33°06′ N 75°40′ E	1459 m.a.s.l
16	Kursari 1, J&K	33°0′ N 75°41′ E	1468 m.a.s.l
17	Kursari 2, J&K	33°0′ N 75°41′ E	1434 m.a.s.l
18	Kursari 3, J&K	33°0′ N 75°41′ E	1459 m.a.s.l
19	Kursari 4, J&K	33°0′ N 75°41′ E	1468 m.a.s.l
20	Khelani, J&K	33°03′ N 75°38′E	1274 m.a.s.l
21	Gatha, J&K	32°59′ N 75°42′ E	1480 m.a.s.l
22	Randa, J&K	32°59′ N 75°43′ E	1583 m.a.s.l
23	Wazir Kotli, J&K	32°58′ N 75°43′ E	1606 m.a.s.l
24	Singhasan Pull, J&K	32°59′ N 75°43′ E	1645 m.a.s.l
25	Kapra, J&K	33°07′ N 75°24′ E	1740 m.a.s.l
26	Powerhouse, J&K	32°56′ N 75°43′ E	1885 m.a.s.l
27	Bhadrote, J&K	32°56′ N 75°43′ E	1898 m.a.s.l
28	MushDev Nallah, J&K	32°56′ N 75°45′ E	1941 m.a.s.l
29	Atalgarh, J&K	32°56′ N 75°45′ E	1941 m.a.s.l
30	Haliyan 1, J&K	32°55′ N 75°42′ E	1774 m.a.s.l
31	Haliyan 2, J&K	32°58′ N 75°42′ E	1706 m.a.s.l
32	Haliyan 3, J&K	33°01′ N 75°41′ E	2664 m.a.s.l
33	Panaja, J&K	32°57′ N 75°43′ E	1763 m.a.s.l
34	Qilla Mohalla, J&K	32°58′ N 75°42′ E	1718 m.a.s.l
35	Almora, Uttrakhand	29°37′ N 79°32′ E	1870 m.a.s.l
36	Chakrata, Uttrakhand	33°33′ N 74°24′ E	1781 m.a.s.l
37	Kund, J&K	33°33′ N 74°23′ E	2159 m.a.s.l
38	Cha, J&K	33°33′ N 74°24′ E	2440 m.a.s.l
39	Tungwali, J&K	33°34′ N 74°24′ E	2858 m.a.s.l
40	Sapanwali, J&K	33°33′ N 74°23′ E	2263 m.a.s.l
41	Azamtabad, J&K	33°33′ N 74°23′ E	2124 m.a.s.l
42	Thajwas, J&K	33°16′ N 75°17′ E	3108 m.a.s.l

m.a.s.l. meters above sea level.

**Table 2:** Pearson’s correlation among nineteen different bioclimatic variables.

Bioclimatic Variables	bio_1	bio_2	bio_3	bio_4	bio_5	bio_6	bio_7	bio_8	bio_9	bio_10	bio_11	bio_12	bio_13	bio_14	bio_15	bio_16	bio_17	bio_18	bio_19
bio_1	1																		
bio_2	0.63																		
bio_3	0.52	0.56																	
bio_4	−0.39	−0.05	−0.78																
bio_5	0.91	0.71	0.24	0															
bio_6	0.94	0.51	0.67	−0.63	0.75														
bio_7	−0.08	0.25	−0.63	0.91	0.31	−0.38													
bio_8	0.46	0.18	0.39	−0.4	0.32	0.53	−0.32												
bio_9	0.92	0.5	0.32	−0.27	0.88	0.84	0.01	0.23											
bio_10	0.92	0.67	0.25	−0.02	0.99	0.77	0.26	0.34	0.88										
bio_11	0.95	0.55	0.68	−0.63	0.76	0.99	−0.35	0.51	0.85	0.78									
bio_12	0.33	−0.26	0.42	−0.7	0	0.48	−0.7	0.41	0.24	0.06	0.48								
bio_13	0.18	−0.28	0.48	−0.74	−0.15	0.39	−0.78	0.38	0.06	−0.1	0.37	0.91							
bio_14	0.04	−0.08	−0.66	0.59	0.27	−0.14	0.59	−0.2	0.2	0.28	−0.14	−0.38	−0.65						
bio_15	0.06	−0.21	0.57	−0.77	−0.25	0.31	−0.81	0.35	−0.06	−0.23	0.29	0.72	0.91	−0.84					
bio_16	0.21	−0.26	0.5	−0.73	−0.12	0.41	−0.77	0.36	0.1	−0.06	0.4	0.93	0.99	−0.63	0.89				
bio_17	0.26	−0.23	−0.36	−0.01	0.27	0.19	0.1	−0.04	0.43	0.26	0.21	0.16	−0.13	0.66	−0.32	−0.12			
bio_18	0.29	−0.06	0.6	−0.68	−0.02	0.44	−0.68	0.48	0.12	0.03	0.45	0.9	0.88	−0.6	0.76	0.9	−0.2		
bio_19	0.32	−0.03	−0.32	0.07	0.38	0.19	0.25	−0.11	0.51	0.36	0.23	0.04	−0.29	0.68	−0.46	−0.26	0.94	−0.25	1

**Table 3:** Bioclimatic variables selected for modeling the distribution of *Valeriana wallichii* in the present study.

Variable	Description
BIO-1	(Annual Mean Temperature)
BIO-2	(Mean Diurnal Range)
BIO-3	(Isothermality)
BIO-7	(Temperature Annual Range)
BIO-8	(Mean Temperature of Wettest Quarter)
BIO-12	(Annual Mean Precipitation)
BIO-14	(Precipitation of Driest Month)
BIO-17	(Precipitation of Driest Quarter)

**Table 4:** Algorithm-wise importance scores of the different bioclimatic variables that were retained for modeling of *Valeriana wallichii*.

Bioclimatic Variable	GLM	GBM	GAM	CTA	ANN	SRE	FDA	RF	MAXENT. Phillips	Mean
bio_01	0.80	0.14	0.66	0.27	0.60	0.48	0.39	0.06	0.69	0.45
bio_02	0.59	0.02	0.70	0.00	0.43	0.34	0.02	0.03	0.27	0.27
bio_03	0.19	0.08	0.61	0.09	0.16	0.21	0.01	0.05	0.12	0.17
bio_07	0.37	0.08	0.65	0.07	0.20	0.19	0.06	0.04	0.29	0.22
bio_08	0.09	0.01	0.41	0.02	0.38	0.22	0.26	0.02	0.54	0.22
bio_12	0.49	0.02	0.65	0.08	0.59	0.34	0.08	0.02	0.49	0.31
bio_14	0.07	0.06	0.55	0.21	0.13	0.23	0.15	0.11	0.30	0.20
bio_17	0.54	0.13	0.82	0.64	0.78	0.36	0.25	0.06	0.57	0.46

**Table 5:** Summary of the range change statistics for *Valeriana wallichii* under climate change scenarios compared to current climatic conditions.

Scenario	Ensemble Type	Loss	Absent	Stable	Gain	Percent Loss	Percent Gain	Range Change (%)
RCP4.5 2050	Committee averaging	22,334	895,491	16,690	6514	57.231	16.692	−40.539
RCP4.5 2070	Committee averaging	34,439	898,663	4585	3342	88.251	8.564	−79.687
RCP8.5 2050	Committee averaging	34,801	898,511	4223	3494	89.178	8.953	−80.225
RCP8.5 2070	Committee averaging	38,196	900,519	828	1486	97.878	3.808	−94.070
RCP4.5 2050	Weighted mean	25,408	885,378	21,537	8706	54.123	18.545	−35.578
RCP4.5 2070	Weighted mean	39,793	889,563	7152	4521	84.765	9.630	−75.135
RCP8.5 2050	Weighted mean	38,494	889,352	8451	4732	81.998	10.080	−71.918
RCP8.5 2070	Weighted mean	45,560	891,102	1385	2982	97.050	6.352	−90.698

**Table 6:** Niche comparisons and first two principal components between current and future projected distribution of the *Valeriana wallichii*.

Pair	PC1 (%)	PC2 (%)	Overlap (D)	Equivalency Test (*p*-Value)	Similarity Test (*p*-Value)
Current vs. RCP 4.5 2050	45.11	33.6	0.66	0.45545	0.0198
Current vs. RCP 4.5 2070	43.08	25.59	0.60	0.55446	0.05941
Current vs. RCP 8.5 2050	45.76	32.77	0.65	0.52475	0.0297
Current vs. RCP 8.5 2070	44.65	33.28	0.42	0.46436	0.0495

## Data Availability

Not applicable.

## Abbreviations

AUC	Area Under Curve
TSS	True Skill Statistics
RCP	Representative Concentration Pathway
PCA	Principal Component Analysis
IUCN	International Union for Conservation of Nature and Natural Resources
ANN	Artifical Neural Network
CTA	Cluster Tree Analysis
GAM	Generalized Additive Model
GBM	Generalized Boosted Model
GLM	Generalized Linear Model
MaxEnt	Maximum Entropy
RF	Random Forest
SRE	Surface Range Envelope
FDA	Flexible Discriminant Analysis

## References

[1] Cruz R.V., Harasawa H., Lal M., Wu S., Anokhin Y., Punsalmaa B., Honda Y., Jafari M., Li C., Huu N.N., Parry M.L., Canziani O.F., Palutikof J.P., van der Linden P.J., Hanson C.E. (2007). Asia climate change 2007: Impacts, adaptation and vulnerability. Contribution of Working Group II to the Fourth Assessment Report of the Intergovernmental Panel on Climate Change.

[2] Shrestha U.B., Gautam S., Bawa K.S. (2012). Widespread Climate Change in the Himalayas and Associated Changes in Local Ecosystems. PLoS ONE.

[3] Chang J., Zhang H., Wang Y., Zhu Y. (2016). Assessing the impact of climate variability and human activities on streamflow variation. Hydr. Earth Syst. Sci..

[4] Wani I.A., Verma S., Kumari P., Charles B., Hashim M.J., El-Serehy H.A. (2021). Ecological assessment and environmental niche modelling of Himalayan rhubarb (*Rheum webbianum* Royle) in northwest Himalaya. PLoS ONE.

[5] Iannella M., Cerasoli F., Alessandro D.P., Console G., Biondi M. (2018). Coupling GIS spatial analysis and Ensemble Niche Modelling to investigate climate change-related threats to the Sicilian pond turtle *Emystrinacris*, an endangered species from the Mediterranean. Peer J..

[6] Wei S.C., Li H.C., Shih H.J., Liu K.F. (2018). Potential impact of climate change and extreme events on slope land hazard—A case study of Xindian watershed in Taiwan. Nat. Hazards Earth Syst. Sci..

[7] Halloy S.R., Mark A.F. (2003). Climate-change effects on alpine plant biodiversity: A New Zealand perspective on quantifying the threat. Arc. Ant. Alp. Res..

[8] Thullier W., Richardson D.M., Pysek P., Midgley G.F., Huges G.O., Rouget M. (2005). Niche-based modelling as a tool for predicting the risk of alien plant invasions at a global scale. Glob. Change Biol..

[9] Shekhar M., Bhardwaj A., Singh S., Ranhotra P.S., Bhattacharyya A., Pal A.K., Roy I., Martín-Torres F.J., Zorzano M.-P. (2017). Himalayan glaciers experienced significant mass loss during later phases of little ice age. Sci. Rep..

[10] Van de Ven C.M., Weiss S.B., Ernst W.G. (2007). Plant species distributions under present conditions and forecasted for warmer climates in an arid mountain range. Earth Interact..

[11] Engler R., Randin C.F., Thuiller W., Dullinger S., Zimmermann N.E., Araujo M.B., Guisan A. (2011). 21st century climate change threatens mountain flora unequally across Europe. Glob. Change Biol..

[12] Tovar C., Arnillas C.A., Cuesta F., Buytaert W. (2013). Diverging Responses of Tropical Andean Biomes under Future Climate Conditions. PLoS ONE.

[13] Cuena-Lombra~na A., Fois M., Fenu G., Cogoni D., Bacchetta G. (2018). The impact of climatic variations on the reproductive success of Gentiana lutea L. in a Mediterranean mountain area. Int. J. Biometeorol..

[14] Ahmed R., Khuroo A.A., Hamid M., Charles B., Rashid I. (2019). Predicting invasion potential and niche dynamics of *Parthenium hysterophorus* (Congress grass) in India under projected climate change. Biodivers. Conserv..

[15] Malhi Y., Franklin J., Seddon N., Solan M., Turner M.G., Field C.B., Knowlton N. (2020). Climate change and ecosystems: Threats, opportunities and solutions. Phil. Trans. R. Soc..

[16] Taleshi H., Jalali S.G., Alavi S.J., Hosseini S.M., Naimi B., Zimmermann N.E. (2019). Climate change impacts on the distribution and diversity of major tree species in the temperate forests of Northern Iran. Reg. Environ. Change.

[17] Guisan A., Thuiller W. (2005). Predicting species distribution: Offering more than simple habitat models. Ecol. Lett..

[18] Bobrowski M., Weidinger J., Schwab N., Schickhoff U. (2021). Searching for ecology in species distribution models in the Himalayas. Ecol. Modell..

[19] Rew J., Cho Y., Moon J., Hwang E. (2020). Habitat Suitability Estimation Using a Two-Stage Ensemble Approach. Remote Sens..

[20] Tingley M., Darling E., Wilcove D. (2014). Fine- and coarse-filter conservation strategies in a time of climate change. Ann. N. Y. Acad. Sci..

[21] Singh L., Tariq K.M., Chandra S., Bhatt I.D., Nandi S.K. (2017). Ecological niche modelling: An important tool for predicting Suitable habitat and conservation of the himalayan medicinal Herbs. Envis Bull. Himal. Ecol..

[22] Guisan A., Zimmermann N.E. (2000). Predictive Habitat distribution models in ecology. Ecol. Modell..

[23] Pacifici M., Foden W.B., Visconti P., Watson J.E.M., Butchart S.H.M., Kovacs K.M., Scheffers B.R., Hole D.G., Martin T.G., Akçakaya H.R. (2015). Assessing species vulnerability to climate change. Nat. Clim. Change.

[24] Hao T., Elith J., Guillera-Arroita G., Lahoz-Monfort J.J., Serra-Diaz J. (2019). A review of evidence about use and performance of species distribution modelling ensembles like BIOMOD. Divers. Distrib..

[25] Cengic M., Rost J., Remenska D., Janse J.H., Huijbregts M.A.J., Schipper A.M. (2020). On the importance of predictor choice, modelling technique, and number of pseudo-absences for bioclimatic envelope model performance. Ecol. Evol..

[26] Condro A.A., Prasetyo L.B., Rushayati S.B., Santikayasa I.P., Iskandar E. (2021). Predicting Hotspots and Prioritizing Protected Areas for Endangered Primate Species in Indonesia under Changing Climate. Biology.

[27] Elith J., Leathwick J.R. (2009). Species Distribution Models: Ecological Explanation and Prediction Across Space and Time. Ann. Rev. Ecol. Evol. Syst..

[28] Gaston K.J. (1996). Species Richness: Measure and Measurement. Biodiversity: A Biology of Numbers and Difference.

[29] Pecl G.T., Araújo M.B., Bell J.D., Blanchard J., Bonebrake T.C., Chen I.C., Williams S.E. (2017). Biodiversity redistribution under climate change: Impacts one ecosystems and human well-being. Science.

[30] Wani I.A., Kumar V., Verma S., Jan A.T., Rather I.A. (2020). *Dactylorhiza hatagirea* D. Don soo: A critically endangered perennial orchid From the North-West Himalayas. Plants.

[31] Wani I.A., Verma S., Ahmad P., El-Serehy H., Hashim M.J. (2022). Reproductive biology of rheum webbianum Royle, A vulnerable medicinal her from the alpines of North Western Himalaya. Front. Plant Sci..

[32] Rodríguez J., Brotons L., Bustamante J., Seoane J. (2007). The application of predictive modeling of species distribution to biodiversity conservation. Divers. Distrib..

[33] Rashid I., Romshoo S.A., Chaturvedi R.K., Ravindranath N.H., Sukumar R., Jayaraman M., Lakshmi T.V., Sharma J. (2015). Projected climate change impacts on vegetation distribution over Kashmir Himalayas. Clim. Chan..

[34] Thuiller W., Lafourcade B., Engler R., Araújo M.B. (2009). BIOMOD—A platform for ensemble forecasting of species distributions. Ecography.

[35] Cianfrani C., Lay G.L., Maiorano L., Satizábal H.F., Loy A., Guisan A. (2011). Adapting global conservation strategies to climate change at the European scale: The otter as a flagship species. Biol. Conserv..

[36] Hastie T., Tibshirani R., Buja A. (1994). Flexible Discriminant Analysis by Optimal Scoring. J. Am. Stat. Assoc..

[37] Kumari P., Khajuria A., Wani I.A., Khan S., Verma S. (2020). Effect of floral size reduction on pollination and reproductive efficiency of female flowers of *Valeriana wallichii*, a threatened medicinal plant. Nat. Acad. Sci. Lett..

[38] Barbet-Massin M., Jiguet F., Albert C.H., Thuiller W. (2012). Selecting pseudo-absences for species distribution models: How, where and how many?. Methods Ecol. Evol..

[39] Guisan A., Thuiller W., Zimmermann N.E. (2017). Habitat Suitability and Distribution Models: With Applications in R.

[40] Allouche O., Tsoar A., Kadmon R. (2006). Assessing the accuracy of species distribution models: Prevalence, kappa and the true skill statistic (TSS). J. Appl. Ecol..

[41] Marmion M., Parviainen M., Luoto M., Heikkinen R.K., Thuiller W. (2009). Evaluation of consensus methods in predictive species distribution modelling. Divers Distrib..

[42] Peterson A.T., Soberón J., Pearson R.G., Anderson R.P., Martínez-Meyer E., Nakamura M., Araújo M.B. (2011). Ecological Niches and Geographic Distributions.

[43] Beaumont L.J., Graham E., Duursma D.E., Wilson P.D., Cabrelli A., Baumgartner J.B., Hallgren W., Laffan S.W. (2016). Which species distribution models are more (or less) likely to project broad-scale, climate induced shifts in species ranges?. Ecol. Modell..

[44] Broennimann O., Fitzpatrick M.C., Pearman P.B. (2012). Measuring ecological niche overlap from occurrence and spatial environmental data. Glob. Ecol. Biogeogr..

[45] Petitpierre B., Kueffer C., Broennimann O., Randin C., Daehler C., Guisan A. (2012). Climatic niche shifts are rare among terrestrial plant invaders. Science.

[46] Warren D.L., Glor R.E., Turelli M. (2008). Environmental Niche Equivalency Versus Conservatism: Quantitative Approaches to Niche Evolution. Evolution.

[47] Di Cola V., Broennimann O., Petitpierre B., Breiner F.T., D’Amen M., Randin C., Engler R., Pottier J., Pio D., Dubuis A. (2017). Ecospat: An R package to support spatial analyses and modelling of species niches and distributions. Ecography.

[48] Zhong Y., Xue Z., Jiang M., Liu B., Wang G. (2021). The application of species distribution modeling in wetland restoration: A case study in the Songnen Plain, Northeast China. Ecol. Indic..

[49] Mushtaq S., Reshi Z.A., Shah M., Charles B. (2021). Modelled distribution of an invasive alien plant species differs at different spatio-temporal scales under changing climate: A case study of *Parthenium hysterophorus* L.. Trop. Ecol..

[50] Davies T.J., Purvis A., Gittleman J.L. (2009). Quaternary climate change and the geographic ranges of mammals. Amer. Natural..

[51] Cardillo M., Mace G.M., Gittleman J.L., Jones K.E., Bielby J., Purvis A. (2008). The predictability of extinction: Biological and external correlates of decline in mammals. Proc. Royal Soc. Biol. Sci..

[52] Purvis A., Gittleman J.L., Cowlishaw G., Mace G.M. (2000). Predicting extinction risk in declining species. Proc. Royal Soc. Biol. Sci..

[53] Bellard C., Bertelsmeier C., Leadley P., Thuiller W., Courchamp F. (2012). Impacts of climate change on the future of biodiversity. Ecol. Lett..

[54] Telwala Y., Brook B.W., Manish K., Pandit M.K. (2013). Climate-Induced Elevational Range Shifts and Increase in Plant Species Richness in a Himalayan Biodiversity Epicentre. PLoS ONE.

[55] Palacios C.R., John C.W. (2020). Recent responses to climate change reveal the drivers of species extinction and survival. Proc. Nat. Acad. Sci. USA.

[56] Tewari V.P., Verma R.K., Von Gadow K. (2017). Climate change effects in the Western Himalayan ecosystems of India: Evidence and strategies. For. Ecosyst..

[57] Sharma A., Dubey V.K., Johnson J.A., Rawal Y.K., Sivakumar K. (2021). Is there always space at the top? Ensemble modeling reveals climate-driven high-altitude squeeze for the vulnerable snow trout *Schizothorax richardsonii* in Himalaya. Ecol. Indic..

[58] Dechen L., Gabriele C., Sommer S., Phuntsho T., Namgay W., Sonam W., Arpat O. (2021). Modeling distribution and habitat suitability for the snow leopard in Bhutan. Front. Conserv. Sci..

[59] Pant G., Maraseni T., Apan A., Allen B.L. (2021). Predicted declines in suitable habitat for greater one-horned rhinoceros (*Rhinoceros unicornis*) under future climate and land use change scenarios. Ecol. Evol..

[60] Amanda M.W., Kumar S., Brown C.S., Stohlgren T.J., Bromberg J. (2016). Field validation of an invasive species Maxent model. Ecol. Inform..

[61] Konowalik K., Nosol A. (2021). Evaluation metrics and validation of presence-only species distribution models based on distributional maps with varying coverage. Sci. Rep..

[62] Wang H.H., Wonkka C.L., Treglia M.L., Grant W.E., Smeins F.E., Rogers W.E. (2020). Species distribution modelling for conservation of an endangered endemic orchid. AoB Plants.

[63] Salam N., Reshi Z., Shah M. (2020). Habitat suitability modelling for *Lagotis cashmeriana* (ROYLE) RUPR., a threatened species endemic to Kashmir Himalayan alpines. Geo. Ecol. Lands.

[64] Ye P., Zhang G., Zhao X., Chen H., Si Q., Wu J. (2021). Potential geographical distribution and environmental explanations of rare and endangered plant species through combined modeling: A case study of Northwest Yunnan, China. Ecol. Evol..

[65] Zangiabadi S., Zaremaivan H., Brotons L., Mostafavi H., Ranjbar H. (2021). Using climatic variables alone overestimate climate change impacts on predicting distribution of an endemic species. PLoS ONE.

[66] Meier E., Kienast F., Pearman P.B., Svenning J.C., Thuiller W., Araújo M.B., Guisan A., Zimmermann N.E. (2010). Biotic and abiotic variables show little redundancy in explaining tree species distributions. Ecography.

[67] Page N.V., Shanker K. (2018). Environment and dispersal influence changes in species composition at different scales in woody plants of the Western Ghats, India. J. Veg. Sci..

[68] Wani I.A., Verma S., Mushtaq S., Alsahli A.A., Alyemeni M.A., Tariq M., Pant S. (2021). Ecological analysis and environmental niche modelling of *Dactylorhiza hatagirea* (D. Don) Soo: A conservation approach for critically endangered medicinal orchid. Saud. J. Biol. Sci..

[69] Chauhan H.K., Bisht A.K., Bhatt I.D., Bhatt A., Gallacher D., Santo A. (2018). Population change of *Trillium govanianum* (Melanthiaceae) amid altered indigenous harvesting practices in the Indian Himalayas. J. Ethnopharmacol..

[70] Tariq M., Nandi S.K., Bhatt I.D., Bhavsar D., Roy A., Pande V. (2021). Phytosociological and niche distribution study of Paris polyphylla Smith, an important medicinal herb of Indian Himalayan region. Trop. Ecol..

[71] Dhyani A., Kadaverugu R., Nautiyal B.P., Nautiyal M.C. (2021). Predicting the potential distribution of a critically endangered medicinal plant *Lilium polyphyllum* in Indian Western Himalayan Region. Reg. Environ. Chang..

[72] Dikshit A., Sarkar R., Pradhan B., Acharya S., Dorji K. (2019). Estimating rainfall thresholds for landslide occurrence in the Bhutan Himalayas. Water.

[73] Ramachandran R., Roy P. (2018). Vegetation response to climate change in Himalayan hill ranges: A remote sensing perspective. Plant Diversity in the Himalaya Hotspot Region.

[74] Hamid M., Khuroo A.A., Charles B., Ahmad R., Singh C.P., Aravind N.A. (2019). Impact of climate change on the distribution range and niche dynamics of Himalayan birch, a typical treeline species in Himalayas. Biodivers. Conserv..

[75] Dimri A.P., Dash S.K. (2012). Wintertime climatic trends in the western Himalayas. Clim. Chang..

[76] Dash S.K., Jenamani R.K., Kalsi S.R., Panda S.K. (2007). Some evidence of climate change in twentieth-century India. Clim. Chang..

[77] Sontakke N.A., Singh H.N., Singh N., Jha M.K. (2009). Monitoring physiographic rainfall variation for sustainable Management of Water Bodies in India. Natural and Anthropogenic Disasters: Vulnerability, Preparedness and Mitigation.

[78] Wei B., Wang R., Hou K., Wang X., Wu W. (2018). Predicting the current and future cultivation regions of *Carthamus tinctorius* L. using MaxEnt model under climate change in China. Glob. Ecol. Conserv..

[79] Parmesan C., Hanley M.E. (2015). Plants and climate change: Complexities and surprises. Ann. Bot..

[80] Fernández M., Hamilton H. (2015). Ecological Niche Transferability Using Invasive Species as a Case Study. PLoS ONE.

[81] Wei J., Zhang H., Zhao W., Zhao Q. (2017). Niche shifts and the potential distribution of *Phenacoccus solenopsis* (Hemiptera: Pseudococcidae) under climate change. PLoS ONE.

[82] Pili A.N., Tingley R., Sy E.Y., Diesmos M.L.L., Diesmos A.C. (2020). Niche shifts and environmental non-equilibrium undermine the usefulness of ecological niche models for invasion risk assessments. Sci. Rep..

